# Variations in cumulative workload and anaerobic power in adolescent elite male football players: associations with biological maturation

**DOI:** 10.1186/s13102-023-00623-5

**Published:** 2023-01-31

**Authors:** Hadi Nobari, Armin Gorouhi, Javier Mallo, Demetrio Lozano, Pablo Prieto-González, Elena Mainer-Pardos

**Affiliations:** 1 Department of Exercise Physiology, Faculty of Educational Sciences and Psychology, University of Mohaghegh Ardabili, Ardabil, 56199‑11367 Iran; 2 Faculty of Sport Sciences, University of Extremadura, 10003 Cáceres, Spain; 3 Department of Motor Performance, Faculty of Physical Education and Mountain Sports, Transilvania University of Braşov, 500068 Braşov, Romania; 4 University of Tor Vergata, Rome, Italy; 5 Facultad de Ciencias de La Actividad Física Y El Deporte (INEF), Universidad Politécnica de Madrid, Madrid, Spain; 6 Health Sciences Faculty, Universidad San Jorge, Autovia A23 Km 299, 50830 Villanueva de Gállego, Saragossa Spain; 7 Health and Physical Education Department, Prince Sultan University, Riyadh, 11586 Saudi Arabia

**Keywords:** Fatigue, Monitoring, Maturity, Peak power, Physical fitness, Football conditioning

## Abstract

**Background:**

It is considered that the maturity condition of young football players is related to their physical performance during short high-intensity efforts dependent on anaerobic power ability. Hence, the aim was to determine possible relationships between maturity status and training intensity by measuring the footballer´s peak height velocity (PHV), maturity offset and anaerobic power.

**Methods:**

Seventeen youth male players of different playing positions participated in the study and performed tests at three different stages of the season (early-, mid- and end-season) All the anthropometric parameters and biomarkers related to maturation were controlled during the season. The training intensity and load was monitored and the anaerobic power of the players was assessed by a running-based anaerobic sprint test (RAST).

**Results:**

The analysis of all the recorded data revealed a positive relationship between PHV and anaerobic power in the early- and end-season stages (*p* < 0.05). Maturity offset and anaerobic power (mean RAST) showed an absolute correlation in early- and end -season stages (r: − 0.39 to 0.91). The multiple linear regression analysis revealed that min RAST was the best predictor for both PHV and maturity offset. The analysis of the training intensity and workloads showed a positive effect on the performance in the fitness test (*p* < 0.05). Additionally, players experienced less fatigue at the end-season stage than at the early-season.

**Conclusions:**

The results show that coaches might benefit from monitoring training loads and the maturity status of the players in order to enhance their physical performance during the season.

## Introduction

Football is reflected in its many different variables, acknowledged as an acyclic modality that can be considered an intermittent activity from the standpoint of movement [1]. In football, there is a complicated bioenergetics demand characterized by using aerobic and anaerobic pathways to deliver energy in various game scenarios [2]. Furthermore, energy requirements depend on playing positions. Midfielders typically cover the highest distances and central defenders the lowest [3].

Short and high-intensity efforts such as repeated sprint ability, change of direction, and explosive movements require anaerobic capacity [4]. The anaerobic capacity variables of the players can be assessed by different standard tests, like a running-based anaerobic sprint test [5]. This manifestation of the anaerobic capacity, with more or less lactic acid accumulation, is related to the maturity condition of adolescent players, which can be evaluated by the maturity offset and peak height velocity (PHV) measurements [6]. Therefore, athletes must be exposed to a progressive increase in the training load over time to develop long-term physical qualities, ensuring an adequate recovery during the phases. To control this development process, qualified professionals should be in charge of elaborating the training plan, considering the individual performance capacity and predispositions (i.e., gender, age, maturation status, anthropometric parameters, recovery/injury status, and force speed profiles) [7].

In this context, fat-free mass is one of young football players' most significant predictors of maximum speed, endurance, and jumping ability [8]. Moreover, performance enhancements also depend on improvements in the nervous system maturation, cardiovascular and lung capacity, anthropometric characteristics, hemoglobin level, and blood volume [9].

To be successful in the game, young football players should develop and maintain a high level of different physical qualities [10], such as anaerobic power, which represents the ability to execute work at maximum speed. Moreover, this explosive movement requires rapid energy production by the alactic anaerobic metabolism (ATP-CP). This energy almost exclusively comes from the high-energy phosphocreatine reserves in the muscles, with each kg of muscle containing from 3 to 8 mmol of ATP [11]. For instance, maximum anaerobic power is a crucial trainable variable for a young football player, considering the high metabolic, physiological, and physical demands. Furthermore, football is a sport where the combination of efforts can be used as a promotion strategy [12].

It vital to train the anaerobic capacity because players constantly perform jumps or sprints with little recovery time during a match [13]. Most sprints in football games cover an average distance between 5 and 30 m and take less than 10 s [14]. Anaerobic power aims to make the player faster and more powerful when performing a short exercise over. Thus, its training produces morphological, metabolic, and functional changes in the athlete. In this context, training programs are crucial to produce intense activities better [15]. As an example, plyometric training has been shown as an interesting strategy to enhance the anaerobic power of football players [16]. Another strategy to improve this determinant factor can be sprint training, which is used to enhance explosive power of the lower body limbs and, in consequence, the anaerobic power of elite adult and young athletes [17]. Additionally, anaerobic power can also be improved during gaming situations and interval training (i.e., sprints with and without the ball during training drills) [18]. In addition, monitoring the training load in football is important to ensure an adequate balance between training and recovery [19]. Therefore, coaches and physical trainers must emphasize and understand the process of these relationships for the correct management and organization of the training program. From all of the above, this study aimed to analyze the relationships between maturation variables (PHV and maturity offset) and the accumulated training workload and anaerobic power performance in elite young football players. Therefore, it is essential to understand the maturation status of the young athlete so their harmonious development is not affected, both as a human being and an athlete. Although anaerobic power is a determinant factor for football performance, this physical fitness component has not been researched much in adolescent players. In addition, previous studies have found that changes of direction, speed, power, and strength are decisive determinants of young performance [20] which it has not been considered as well as possible in adolescent players. Even though there are similarities in movement patterns when playing football across different age groups [21, 22] there are several factors such as growth, maturation, training status, and age that need to be addressed when comparing young and adult players, as they have an impact on performance. So through this study, the role of these factors on comprehending the relationships between variations in accumulated workloads and anaerobic power in elite young football players may become clear [23]. Moreover, it is hypothesized that the maturity condition of young football players could affect their physical performance during repeated sprint ability efforts dependent on anaerobic capacity.

## Materials and methods

### Participants

A total of 17 male football players aged 15 to 16 volunteered to take part in this study after providing informed consent. (height: 170.2 ± 8.4 cm; weight: 58.2 ± 6.2 kg). The sample was divided by the players' playing positions, and their characteristics are shown in Table 1. The ethics committee of the Institutional Review Board of the University of Mohaghegh Ardabili approved all procedures for the use of human participants in accordance with the most recent version of the Helsinki Declaration. Prior to the start of the study, written informed parental consent and participant assent were obtained. Before the experiment began, all participants and their parents/legal representatives were fully informed of the protocol's potential risks and benefits.

**Table 1:** Youth football players' body composition and maturation characteristics by playing position. Median ± Standard deviation

Playing position	Age (years)	Height (cm)	Body mass (kg)	PHV (years)	Maturity offset (years)
Defenders (n = 6)	15.6 ± 0.2	172.9 ± 8.4	60.8 ± 4.5	1.4 ± 0.4	14.2 ± 0.5
Wingers (n = 3)	15.6 ± 0.5	163 ± 4.1	54.7 ± 4.1	0.5 ± 0.5	14.5 ± 0
Forwards (n = 2)	15.4 ± 0	170.8 ± 15.9	55.5 ± 10.6	0.7 ± 1.3	14.7 ± 1.3
Central midfielders (n = 6)	15.6 ± 0.2	172.1 ± 6.5	59.9 ± 6.1	0.5 ± 0.5	14.2 ± 0.5

PHV, peak height velocity

### Procedures

A descriptive longitudinal study with a cohort was designed to monitor the daily training workload in an U16 football team during the 15-week competitive season: Early-season (EaS) weeks (W) W1 to W5; mid-season (MiS) W6 to W10; and end-season (EnS) W11 to W15. This design allowed for comparisons between stages, both with and without taking the players' playing positions into account.

### Material and testing

The players were evaluated on two consecutive days in each stage. During the first day, the participants' anthropometric measurements and body composition (i.e., height, sitting height, weight) were evaluated to calculate the maturation status (PHV and maturity offset). The players took an anaerobic power test on the second day. All the evaluations were performed simultaneously and at the same temperature in an artificial football field. All players were previously familiarized with the anaerobic power test and used football boots.

#### Anthropometry and maturity

All anthropometric and body composition measurements were performed during the morning. The subject’s height and sitting height was measured by a skilled person using a stadiometer (Seca model 213, Germany) with a precision of 5 mm. The body weight was measured and recorded with a digital scale (Seca model 813, UK) to the nearest 0.1 kg. The maturity o set and age at PHV were calculated using the Mirwald formula and the information gathered above [24]: maturity offset =  − 9.236 + 0.0002708 (leg length × sitting height) − 0.001663 (age × leg length) + 0.007216 (age × sitting height) + 0.02292 (weight by height ratio), where R = 0.94, R2 = 0.891, and SEE = 0.592). Subtracting the sitting height from the standing height revealed the leg length.

#### Training workloads monitoring

Thirty minutes following the end of each training session, a Category-Ration-10 Borg RPE-scale was used to evaluate the intensity of training [25]. One represents a relatively easy training session on this scale, while 10 represents a highly intense training session. The total training workload (WL) was calculated by multiplying the s-RPE by the training time (in minutes) or each training session and was expressed in arbitrary units. These data were used to analyze weekly workload parameters: Acute (wAWL), Chronic (wCWL), Acute:Chronic (wACWLR), training monotony (wTM), and training strain (wTS) [26, 27]. After the third and fourth weeks, the wCWL and wACWLR were determined using the uncoupled formula, respectively.

#### Anaerobic power test

The running-based anaerobic sprint test (RAST) test was used to assess anaerobic power [28]. Sprinting times were measured with photo-electric timing gates (Newtest Powertimer 300-series device made in Finland). The starting position of the players was adopted following the guidance of a modified 5-0-5 test [27, 29]. Each player performed six repetitions of 35 m sprints at their maximum speed, with a 10 s recovery between each repetition. Once the players completed the test, the following variables were obtained: peak power (RASTpeak), minimum power (RASTmin); average power (RASTave), and fatigue (Fatiguein) = which was calculated using the following formula (RASTpeak − RASTmin)/total time to cover the six sprints. The test–retest intra-class coefficient was 0.89 for this test.

### Statistical analysis

Statistical analyses were performed using the Statistical Package for Social Sciences (SPSS version 23.0, IBM SPSS Inc. Chicago, IL, United States) and GraphPad Prism 8.0.1 (GraphPad Software Inc, San Diego, California, USA). The normality of the distribution of the data was examined with the Shapiro–Wilk test. Pearson and Spearman correlation analysis were performed between the anaerobic power variables and both maturation variables (PHV and maturity offset). A repeated-measures analysis of variance (ANOVA) was used to compare the differences between the three mesocycles, followed by the Bonferroni post-hoc test for pairwise comparisons. For the repeated measure ANOVA, the effect size was calculated using Partial eta squared (ηp^2^). The standardized mean difference (Cohen’s *d*), which represents ESs, is shown along with 95% confidence intervals (CI). The ESs were interpreted using Hopkins et al. guideline’s for standardized mean difference to determine the amount of pairwise comparisons between meso-cycles [30].

The percentage of anaerobic power parameters with differences in WL parameters and maturity variables were analyzed using multiple linear regression. To corroborate inferences regarding each model's adequacy, the Akaike information criterion (AIC) for the regression for each model was also calculated.

## Results

In Table 2 significant positive correlations (r = 0.42 to 0.54; *p* ≤ 0.05) were shown between PHV with RASTpeak early-season, RASTpeak end-season, RASTave early-season and RASTave end-season. In addition, RASTmin early-season was associated (r = 0.66 to 0.94; *p* ≤ 0.05) with RAST min end-season, RASTpeak early-season, RASTpeak end-season, RASTave early-season and RASTave2. There were associations (r = -0.90 to 0.92; *p* ≤ 0.01) between RASTmin end-season and RASTpeak early-season, RASTpeak end-season, RASTave early-season, and RASTave end-season. Furthermore, RAST peak early-season was associated (r = 0.69 to 0.96; *p* ≤ 0.01) with RASTpeak end-season, RASTave early-season, RASTave end-season, Fatiguein early-season, and Fatiguein end-season. Additionally, RASTpeak end-season was associated (r = 0.77 to 0.89; *p* ≤ 0.05) with RASTave early-season, RASTave2, Fatiguein early-season and Fatiguein end-season. There were relations (r = 0.30 to 0.98; *p* ≤ 0.05) between RASTave early-season and RASTave end-season, Fatiguein early-season and Fatiguein end-season. RASTave end-season was associated (r = 0.48 to 0.93; *p* ≤ 0.05) with Fatiguein early-season and Fatiguein end-season. Finally, Fatiguein early-season was related to Fatiguein end-season (r = 0.93; *p* ≤ 0.01).

**Table 2:** Pearson and Spearman correlation analysis between the peak height velocity and anaerobic power indexes

Variable	β0	β1	β2	β3	β4	β5	β6	β7	β8
PHV (β0)	1								
RAST_min_1 (β1)	0.27	1							
RAST_min_2 (β2)	0.38	0.94**	1						
RAST_peak_1 (β3)	0.42*	0.74**	0.79**	1					
RAST_peak_2 (β4)	0.45*	0.66**	0.75**	0.96**	1				
RAST_ave_1 (β5)	0.48*	0.88**	0.92**	0.87**	0.85**	1			
RAST_ave_2 (β6)	0.54*	0.80**	0.90**	0.89**	0.89**	0.98**	1		
Fatigue_in_1 (β7)	0.31	0.11	0.24	0.74**	0.77**	0.43*	0.51*	1	
Fatigue_in_2 (β8)	0.27	0.10	0.17	0.69**	0.78**	0.39*	0.48*	0.93**	1

PHV, Peak height velocity; 1: early-season; 2: end-season; RASTmin: RAST minimum; RASTave: RAST average; Fatiguein: Fatigue index; *p < 0.05; **p < 0.01

In the correlations between maturity offset with anaerobic power parameters (Table 3), the most important of them was maturity offset is related (r = − 0.43 to − 0.39; *p* ≤ 0.05) to RASTave early-season and RASTave end-season. Also, RASTmi early-season was associated (r = 0.66 to 0.94; *p* ≤ 0.01) with RAST min end-season (r = 0.94; *p* ≤ 0.01), RASTpeak early-season (r = 0.73; *p* ≤ 0.01), RASTpeak end-season, RASTave early-season and RASTav end-season. There were associations (r = 0.74 to 0.91; *p* ≤ 0.01) between RASTmin end-season and RASTpeak early-season, RASTpeak end-season, RASTave early-season, and RASTave end-season. Moreover, RAST peak early-season was associated (r = 0.69 to 0.96; *p* ≤ 0.01) with RASTpeak end-season, RASTave early-season, RASTave end-season, Fatiguein early-season, and Fatiguein end-season. Furthermore, RASTpeak end-season was related (r = 0.77 to 0.85; *p* ≤ 0.01) to RASTave early-season, RASTave end-season, Fatiguein early-season and Fatiguein end-season. In addition, RASTave early-season was associated (r = 0.39 to 0.97; *p* ≤ 0.01) with RASTave end-season, Fatiguein early-season and Fatiguein end-season. There were relations (r = 0.47 to 0.51; *p* ≤ 0.05) between RASTave end-season and Fatiguein early-season and Fatiguein end-season. Finally, Fatiguein early-season was related to Fatiguein end-season (r = 0.92; *p* ≤ 0.01).

**Table 3:** Analysis of Pearson and Spearman correlations between maturity offset and anaerobic power indexes

Variable	β0	β1	β2	β3	β4	β5	β6	β7	β8
Maturity offset (β0)	1								
RAST_min_1 (β1)	− 0.18	1							
RAST_min_2 (β2)	− 0.30	0.94**	1						
RAST_peak_1 (β3)	− 0.28	0.73**	0.78**	1					
RAST_peak_2 (β4)	− 0.30	0.66*	0.74**	0.96**	1				
RAST_ave_1 (β5)	− 0.39*	0.87**	0.91**	0.87**	0.85**	1			
RAST_ave_2 (β6)	− 0.43*	0.80**	0.90**	0.88**	0.89**	0.97**	1		
Fatigue_in_1 (β7)	− 0.19	0.11	0.24	0.75**	0.77**	0.43*	0.51*	1	
Fatigue_in_2 (β8)	− 0.13	0.10	0.17	0.69**	0.78**	0.39*	0.47*	0.92**	1

1: early-season; 2: end-season; RASTmin: RAST minimum; RASTave: RAST average; Fatiguein: Fatigue index; *p < 0.05; **p < 0.01

**Fig. 1:**
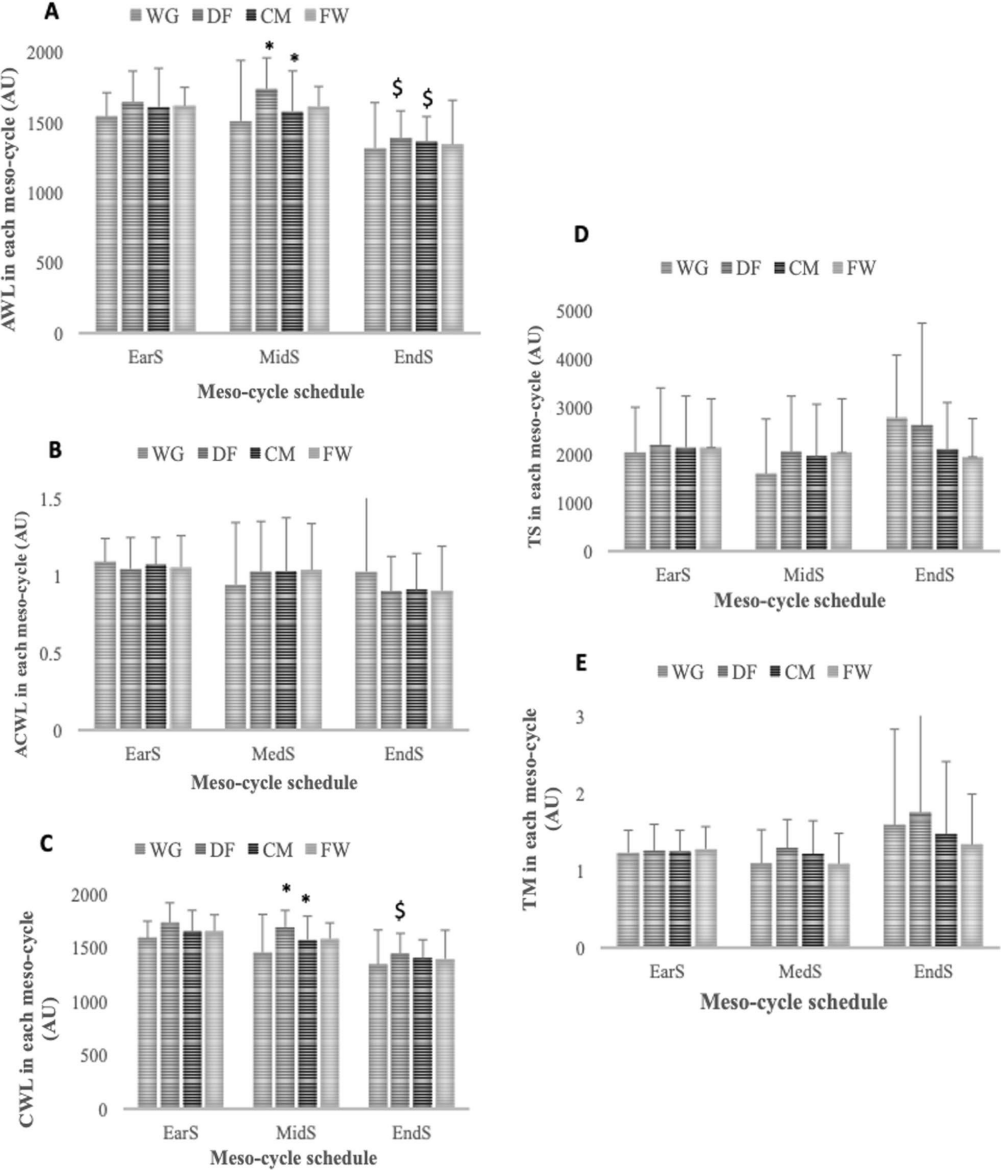
Weekly acute (**A**); chronic workload (**B**); weekly acute-to-chronic workload ratio (**C**); weekly training monotony (**D**), and training stain (**E**) meso-cycle patterns and comparisons over each period during a competition season considering field position and whole team. * represents a statistically significant difference comparing with Ear-S (*p* < 0.05); $ represents a statistically significant difference comparing with Mid-S (*p* < 0.05); AU. arbitrary units; WD. wide defenders; WG: wingers; DF. defenders; CM. central midfielders; FW: forwards; EarS: early-season; MidS: mid-season; EndS: end-season; AWL. weekly acute workload; CWL. weekly chronic workload; ACWLR. weekly acute-to-chronic workload ratio; TM: weekly training monotony; TS: weekly training strain

The highest and the lowest recorded values were observed with regard to wAWL in mid-season [(DF, 1742.1 ± 221.8 arbitrary units (A.U)] and end-season (WG, 1320.1 ± 324.7 A.U), regarding wCWL in early-season (DF, 1732.8 ± 184.1 A.U) and end-season (FW, 1396.3 ± 265.9 A.U), with regard to wACWLR in early-season (WG, 1.08 ± 0.15 A.U) and end-season (DF, 0.89 ± 0.22 A.U), with regard to wTM in end-season (DF, 1.75 ± 2.10 A.U) and mid-season (FW, 1.09 ± 0.39 A.U) and with regard to wTS in end-season (WG, 2774.3 ± 1313.7 A.U) and mid-season (WG, 1615–4 ± 1141.3 A.U). Finally, no significant differences were found between two field positions over the same season period for any periods or workload variables, according to the results (Fig. 1).

Findings from repeated-measure tests ANOVA demonstrated variations in wAWL during the mesocycles of the competition season (*p* =  < 0.001, ηp^2^ = 0.487), wCWL (*p* = 0.002, ηp^2^ = 0.631), wACWLR (*p* = 0.006, ηp^2^ = 0.541), wTM (*p* = 0.01, ηp^2^ = 0.487) and wTS (*p* = 0.04, ηp^2^ = 0.403) (Table 4). Eight comparisons present significant differences in wAWL, wCWL, wACWLR, wTM and wTS from early- to mid-season, wACWL and wCWL from mid- and end-season and wTM from early- to end-season.

**Table 4:** Comparison over competition season meso-cycles in terms of training workload variables

Variables	Season period	Mean ± SD	Comparison	Mean difference (95% CI)	*p*	Effect size (95% CI)
wAWL (AU)	EarS	1281.4 ± 34.7	EarS versus MidS	− 318.1 (− 478.9; − 157.3)	< 0.01*	0.14 (− 0.57; 0.85)
	MidS	1599.5 ± 37.6	EarS versus EndS	− 61.1 (− 189.7; 67.6)	0.65	− 1.92 (− 2.49; − 1.34)
	EndS	1342 ± 39.9	MidS versus EndS	257.1 (124.1; 389.9)	< 0.01*	− 1.64 (− 2.16; − 1.13)
wCWL (AU)	EarS	1320.2 ± 37.1	EarS versus MidS	− 257.1 (− 420.2; − 83.8)	0.01*	− 0.39 (− 1.08; 0.30)
	MidS	1577.2 ± 40.4	EarS versus EndS	− 78.1 (− 223.6; 67.3)	0.50	− 1.86 (− 2.44; − 1.29)
	EndS	1398.3 ± 44.6	MidS versus EndS	178.9 (62.9; 294.8)	0.01*	− 1.13 (− 1.54; − 0.72)
wACWLR (AU)	EarS	0.85 ± 0.02	EarS versus MidS	− 0.09 (− 0.18; − 0.10)	0.02*	− 0.72 (− 1.48; 0.04)
	MidS	0.95 ± 0.02	EarS versus EndS	− 0.09 (− 0.21; 0.03)	0.15	− 1.90 (− 2.83; − 0.97)
	EndS	0.95 ± 0.04	MidS versus EndS	0.01 (− 0.15; 0.16)	> 0.99	− 0.85 (− 1.87; 0.18)
wTM (AU)	EarS	0.957 ± 0.09	EarS versus MidS	− 0.27 (− 0.55; − 0.01)	0.04*	− 0.26 (− 1.08; 0.56)
	MidS	1.234 ± 0.04	EarS versus EndS	− 0.58 (− 1.05; − 0.11)	0.01*	2.21 (1.08; 3.35)
	EndS	0.139 ± 0.13	MidS versus EndS	− 0.30 (− 0.67; 0.06)	0.12	− 1.03 (− 1.43; − 0.63)
wTS (AU)	EarS	1590.1 ± 187.4	EarS versus MidS	− 545.3 (− 1062.3; − 28.4)	0.03*	0.18 (− 0.45; 0.81)
	MidS	2135.4 ± 109.5	EarS versus EndS	− 375.7 (− 970.6; 219.1)	0.31	− 0.24 (− 0.82; 0.34)
	EndS	1965.8 ± 148.7	MidS versus EndS	169.5 (− 234.9; 574.1)	0.81	0.26 (− 0.31; 0.83)

AU, arbitrary units; CI, confidence interval; wAWL, weekly average acute workload in AU; wCWL, weekly average chronic workload in AU; wACWLR, weekly average acute:chronic workload ratio; wTM, weekly average training monotony in AU; wTS, weekly average training strain in AU; Ear-S, early-season period; Mid-S, mid-season period; End-S, end-season period; * Significant differences *p* < 0.05

**Fig. 2:**
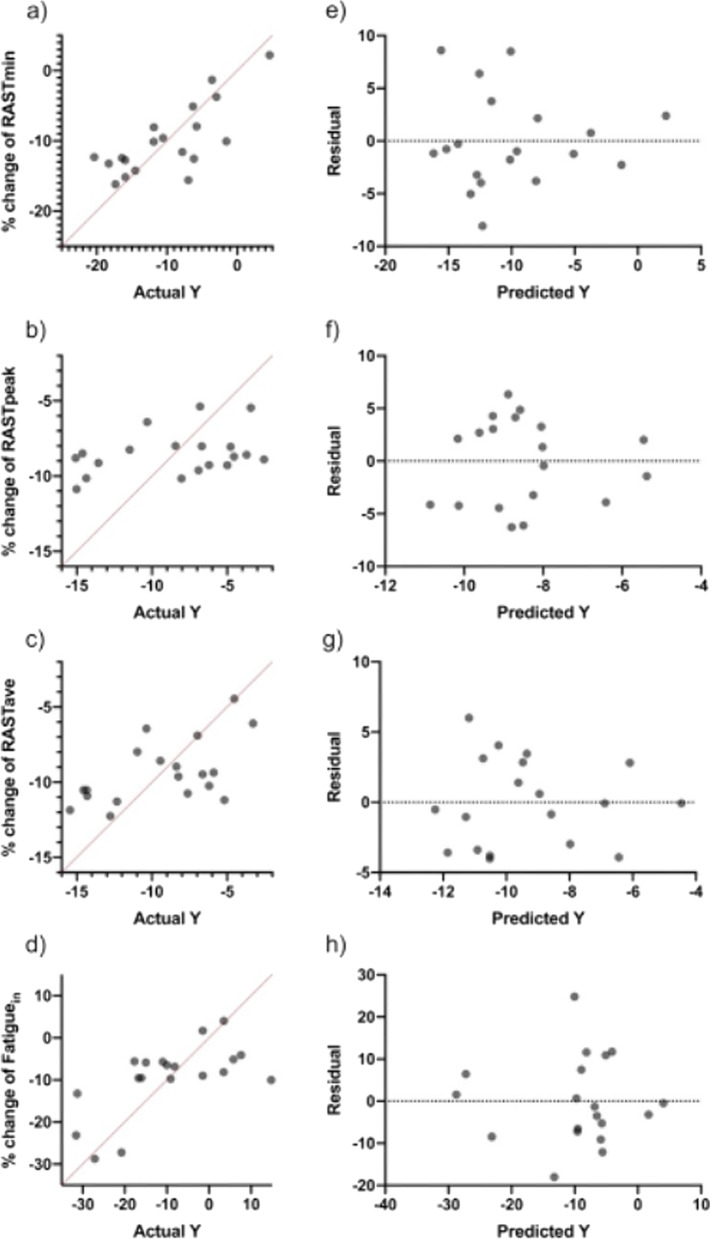
Multiple linear regression analysis was calculated to predict the percentage of change in fitness levels **a** RASTmin. **b** RASTpeak. **c** RASTave and **d** Fatiguein based on accumulated workloads and PHV in the football players. Also. residual plot was calculated to predict the percentage of change in **e** RASTmin. **f** RASTpeak. **g** RASTave and **h** Fatiguein levels; the difference between the actual value of the dependent variable and the value predicted by the residual provided. PHV = Peak height velocity RASTmin: RAST minimum; RASTave: RAST average; Fatigue_in_: Fatigue index

In order to predict the % change in anaerobic power parameters depending on workload and PHV, multiple linear regression analyses were carried out (Table 5 and Fig. 2). RASTmin analysis revealed significant (F (4, 14) = 4.39, *p* = 0.01), with a R2 of 0.55. Participants showed good predictions for RASTmin; (Y) is equal to Beta0 +  + Beta1 (CWL) + Beta2 (ACWLR) + Beta3 (TM) + Beta4 (PHV). RASTave analysis revealed significant (F (2, 16) = 3.75, *p* = 0.04), with a R2 of 0.23. Participants showed good predictions for RASTave; (Y) is equal to Beta0 + Beta1 (ACWLR) + Beta2 (PHV). Fatigue in analysis revealed significant (F (3, 15) = 3.46, *p* = 0.04), with a R2 of 0.29. Participants showed good predictions for Fatigue in; (Y) is equal to Beta0 + Beta1 (AWL) + Beta2 (ACWLR) + Beta3 (TM). Additionally, the analysis in RASTpeak demonstrated there was no significant (F (2, 16) = 1.03, *p* = 0.37) with R2 of 0.11.

**Table 5:** Multiple linear regression analysis: percentage of change in anaerobic power with anaerobic power index. workload parameter and PHV

Variables	Beta	Estimate	|t|	*p* value	95% CI for Estimated	Total predict
RASTmin (%)	β0	25.62	2.32	0.03	2.02. 49.2	**R**^**2**^: 0.55 Estimated** R**^**2**^: 0.43 ***p***: 0.01 **AIC** value: 74.7
CWL (A.U.)	β1	− 0.01	1.25	0.22	− 0.01. 0.01	
ACWLR (A.U.)	β2	− 2.47	1.82	0.08	− 5.37. 0.43	
TM (A.U.)	β3	1.11	1.64	0.12	− 0.33. 2.56	
PHV (years)	β4	− 3.22	1.74	0.10	− 7.18. 0.74	
RASTpeak (%)	β0	− 2.81	0.52	0.60	− 14.1. 8.52	**R**^**2**^: 0.11 Estimated** R**^**2**^: 0.01 ***p***: 0.37 **AIC** value: 63.1
TS (A.U.)	β1	− 0.01	0.71	0.48	− 0.01. 0.01	
PHV (years)	β2	− 1.72	1.12	0.27	− 4.96. 1.51	
RASTave (%)	β0	4.66	0.65	0.52	− 10.3. 19.7	**R**^**2**^: 0.31 Estimated** R**^**2**^: 0.23 ***p***: 0.04 **AIC** value: 52.9
ACWLR (A.U.)	β1	− 0.96	1.59	0.13	− 2.24. 0.31	
PHV (years)	β2	− 2.50	2.16	0.04	− 4.95 − 0.05	
Fatigue_in_ (%)	β0	− 54.4	2.11	0.05	− 109.4. 0.57	**R**^**2**^: 0.40 Estimated** R**^**2**^: 0.29 ***p***: 0.04 **AIC** value: 101.9

AWL, the accumulated acute workload in the season; CWL, the accumulated chronic workload in the season; ACWLR, the accumulated acute: chronic workload ration in the season; TM, the accumulated training monotony in the season; TS, the accumulated training strain in the season; PHV, peak height velocity; %, the percentage of change in between assessments from early-season to after-season; AIC: Akaike information criterion. and CI, confidence interval; VO2max: maximum rate of oxygen consumption.; RASTmin: RAST minimum; RASTave: RAST average; Fatiguein: Fatigue index

**Fig. 3:**
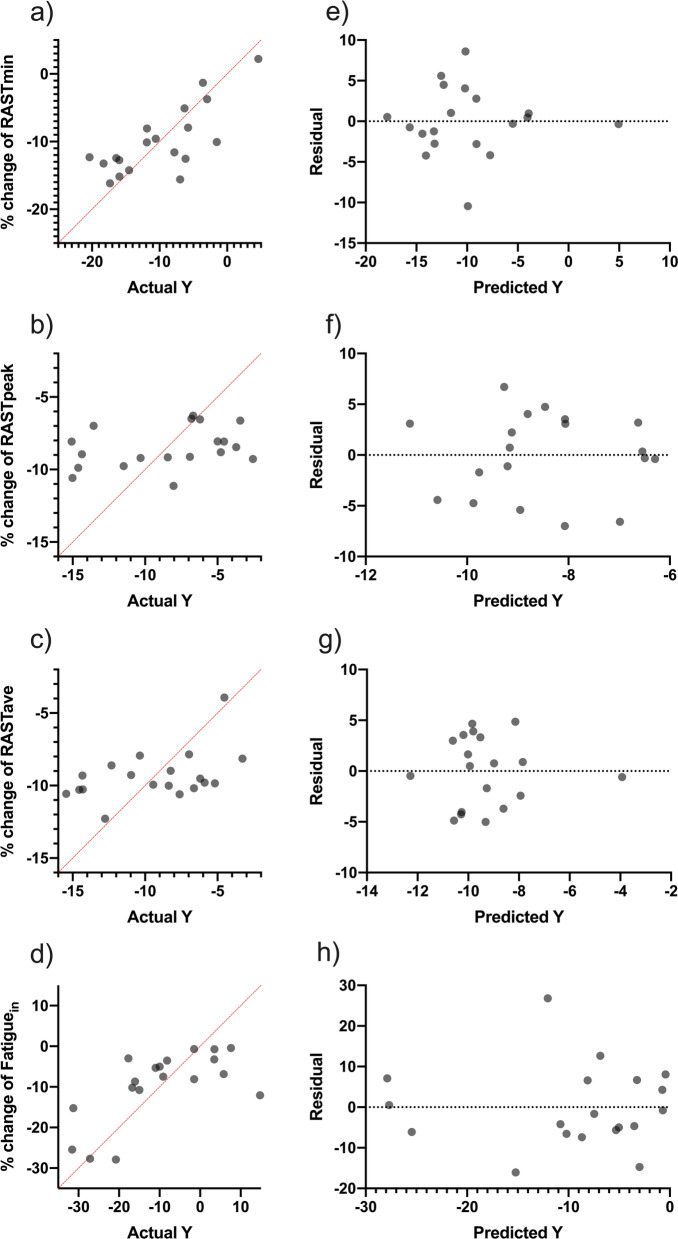
Multiple linear regression analysis was calculated to predict the percentage of change in fitness levels levels **a** RAST_min_. **b** RAST_peak_. **c** RAST_ave_ and **d** Fatigue_in_ based on accumulated workloads and maturity off set in the football players. Also. residual plot was calculated to predict the percentage of change in **e** RAST_min_. **f** RAST_peak_. **g** RAST_ave_ and **h** Fatigue_in_ levels; the difference between the actual value of the dependent variable and the value predicted by the residual provided. Note: RASTmin: RAST minimum; RASTave: RAST average; Fatigue_in_: Fatigue index

In order to predict the % change in anaerobic power parameters depending on workload and maturity offset, multiple linear regression analyses were carried out (Table 6 and Fig. 3). RASTmin analysis revealed significant (F (5, 13) = 4.01, *p* = 0.02), with a R2 of 0.60. Participants showed good predictions for RASTmin; (Y) is equal to Beta0 +  + Beta1 (AWL) + Beta2 (ACWLR) + Beta3 (CWL) + Beta4 (TS) + Beta5 (Maturity offset). Fatigue in analysis revealed significant (F (4, 14) = 2.64, *p* = 0.04), with a R2 of 0.43. Par-ticipants showed good predictions for Fatigue in; (Y) is equal to Beta0 + Beta1 (AWL) + Beta2 (ACWLR) + Beta3 (TS). Additionally, RASTpeak and RASTave analysis revealed no significant (F (2, 16) = 0.96, *p* = 0.40 and F (2, 16) = 2.01, *p* = 0.16) with R2 of 0.20.

**Table 6:** Multiple linear regression analysis: percentage of change in anaerobic power with anaerobic power index. workload parameter and maturity offset

Variables	Beta	Estimate	|t|	*p* value	95% CI for Estimated	Total predict
RASTmin (%)	β0	10.6	0.27	0.78	− 73.3. 94.3	**R**^**2**^: 0.60 Estimated** R**^**2**^: 0.45 ***p***: 0.02 **AIC** value: 77.6
AWL (A.U.)	β1	0.01	1.38	0.19	− 0.01. 0.01	
ACWLR (A.U.)	β2	− 4.10	1.80	0.09	− 9.01. 0.80	
CWL (A.U.)	β3	0.01	1.73	0.07	− 0.02. 0.01	
TS (A.U.)	β4	0.01	1.95	0.07	− 0.01. 0.01	
Maturity offset (years)	β5	3.52	1.78	0.09	− 0.75. 7.79	
RASTpeak (%)	β0	4.60	0.47	0.64	− 15.9.25.1	**R**^**2**^: 0.11 Estimated** R**^**2**^: − 0.01 ***p***: 0.40 **AIC** value: 63.2
AWL (A.U.)	β1	0.01	1.12	0.27	− 0.01. 0.01	
CWL (A.U.)	β2	− 0.01	1.21	0.24	− 0.01. 0.01	
RASTave (%)	β0	23.7	1.11	0.28	− 69.2. 21.6	**R**^**2**^: 0.20 Estimated** R**^**2**^: 0.10 ***p***: 0.16 **AIC** value: 55.9
CWL (A.U.)	β1	− 0.01	1.50	0.15	− 0.01. 0.01	
Maturity offset (years)	β2	1.63	1.17	0.25	− 1.32. 4.59	
Fatigue_in_ (%)	β0	− 55.3	2.21	0.04	− 108.5. − 2.12	**R**^**2**^: 0.43 Estimated** R**^**2**^: 0.31 ***p***: 0.03 **AIC** value: 101.2

AWL, the accumulated acute workload in the season; CWL, the accumulated chronic workload in the season; ACWLR, the accumulated acute: chronic workload ration in the season; TM, the accumulated training monotony in the season; TS, the accumulated training strain in the season; %, the percentage of change in between assessments from early-season to after-season; AIC: Akaike information criterion. and CI, confidence interval; VO_2max_: maximum rate of oxygen consumption.; RASTmin: RAST minimum; RASTave: RAST average; Fatiguein: Fatigue index

## Discussion

This investigation examined the relationship between maturity variables, anaerobic power, and training workloads in young elite football players. The current study's primary evidence revealed a correlation between PHV, anaerobic power, maturity offset, and RASTave. In addition, as the workload progressed, the player's physical performance and resistance against fatigue improved. Another interesting result was that the weekly average workload was higher for defenders in the mid-season and lower for wingers in the end-season period. Moreover, the workload increase is not linear during the season, and the highest values were observed in the mid-season. Finally, different workload parameters explained a good prediction (R2 equal to 0.60) of the difference between anaerobic power (RASTmin) and maturity offset. Anaerobic capacity is characterized by repeated short, high-intensity activities which incorporates acceleration, maximum speed, and agility [31]. The most decisive skills in football, such as performing a jump, sprinting, or scoring a goal, are related to the anaerobic system [32]. Malina et al. [33] introduced the study of anthropometric characteristics (height and weight) as the main factor influencing sprinting in male football players. In addition, Méndez Villanueva et al. [34] showed that maturation played a decisive role in the speed capacity of young male football players. To date, most of the previous research has been focused on aerobic capacity and its improvement from early- to end-season, by assessing variations in VO2max and other aerobic biomarkers. In the current research, a different strategy was adopted to examine how maturity condition and anaerobic performance were related in young football players.

The results of the present study show a positive relationship between PHV and anaerobic power in early- and end-season stages. Similar articles analyzing aerobic and speed variables, a strong relationship was observed between PHV and VO2max [31]. In addition, Clemente et al. [35] showed how speed progressed during the year, with significant developments achieved at the end of the season. On the other hand, Dragijsky et al. [36] showed endurance improvements only in the early-season, whereas performance was reduced in the mid- and end-season evaluations.

The data analysis revealed a significant correlation between maturity offset and RAST average in the early- and end-season. In all these procedures, progression in scores from early- to end-season occurred. In the case of PHV and RAST score calculation, the anaerobic power improvement was demonstrated through RASTmin early-season and end-season. This development also was seen in comparing RASTpeak early-season and end-season. Therefore, there was a relationship between their average. These investigations are important to understand the role of workload and its progression in each period of the season. In a similar study each year observed, there was a rise in levels of all accumulated TM of PHV, the reason for this relationship between PHV and accumulated TM is reported in the differences in CWL [27]. The peak height velocity is introduced as a somatic maturation, and it has a strong relationship with physical performances and cardiac or neuro maturations [37]. In this context, Philippaerts et al. [38] showed that the most progression at PHV on several physical fitness elements is necessary for physical abilities such as anaerobic capacity and running speed.

Moreover, on the other tests which researched the maturity offset and anaerobic power, the association between RASTmin early-season and RASTmin end-season was evident. Despite this, it would be suitable to only consider the RASTave in early- and end-season because their extreme relationship could show other correlations in minimum and peak tests. It has been stated that biological maturation is a gradual process that occurs over time, the improvement of hormonal circulation level leads to neural function, co-ordination, and rising power in both oxidative and non-oxidative physical abilities, and lastly, this metabolism alteration influences players’ performances [39].

After analyzing the results of fatigue in the early and end-season, their correlations with RAST early- and end-season and the reduction in fatigue end-season after RAST end-season compared with fatigue early-season after RAST early-season were observed. These results showed that physical readiness and an increased player resistance against fatigue occurred regarding the workload progression. Physical fitness of football players is measured by monitoring, and it is believed to improve during the season through an applied training load [40]. This feature is essential to get an overview of the body condition of players after each session and set next the training based on acquired information [31].

Based on various types of weekly workload tests concentration such as acute, chronic, training monotony, and training strain, it has been investigated if defenders had higher values in weekly average workload in the mid-season period from W6 to W10 and the lowest values of this test were related to wingers in the end-season from W11 to W15. After collecting the test results, it has been demonstrated that defenders also had the highest value at end-season for the weekly training monotony test. It should be noted that the wingers´ value for weekly training strain was the highest one in mid-season and the lowest one in end-season. In a similar study, the impact of maturation on players’ performances was reported mainly in forward players [31]. According to the examination of all the tests in three different periods of the season (and then separating the scores of players by referring to their positions in the field), it was observed that the workload increases from early- to end- season is not linear. By proper consideration, the highest values were collected in the mid-season. Furthermore, end-season values were higher than the same tests values in the early-season. This result confirmed the previous study showing the highest values of wAWL, wCWL, and wTS in the mid-season and the lowest values in the early-season [41].

The multiple linear regression shows the difference of values and their predictions between anaerobic power and the workload with maturity offset and PHV. According to statistical analysis of the scores, RASTmin, which is included the CWL, ACWLR, TM introduced as the best values through to the participant’s predictions for PHV with R2 of 0.55 total. The comparison between anaerobic power and maturity offset shows the RASTmin scores, which consist of the AWL, CWL, ACWLR, and TS, was as same as the PHV, and players had great predictions in this test with R2 0.60 total. It is worth noting that the RAST peak and RAST average values did not show any positive correlation in comparison with their predicted values. In a similar article, the multiple linear regression was considered on the anaerobic variable workload parameters and PHV of football players, participants showed poor predictions in most elements, such as change of direction and CWL. No significant correlations were recorded in these cases. However, the main results acquired include ACWLR with 0.78 R2 and TM with 0.81 R2 [27]. In another study, the multiple linear regression was used to examine fitness changes based on VO2max along with peak power and maturity status of elite youth players. Participants had good predictions for VO2max with 0.55 R2, and also for peak power (R2 = 0.63) [31].

The main limitation of this study was the number of participants. Working on a higher number of players may reach more accurate results. However, the tests' sensitivity and difficulty were the reasons why some players were not interested in participating. Moreover, if the players’ performances were controlled through external monitoring, for instance, through GPS features, the measurements would be more precise. Ultimately, maturation status was not assessed by using skeletal age. It is therefore recommended that future studies consider the inclusion of the maturational status and the parameters related to this variable. In the future studies, it would be possible to consider PHV and maturity offset and their effects on the performance of other types of trainings such as coordination, balance, decision making and reaction time of young football players according to the direct effect of growth and maturity process of neuro system on these types of activities.

## Conclusions

Anthropometric parameters and maturity status have a strong correlation with anaerobic performance in young soccer players. Therefore, players can attain additional improvements in the anaerobic power performance during the season if their coaches dose the workloads taking into account their peak height velocity and especially their maturity offset. The prescription and application of an adequate training dose in each of the mesocycles might enhance positive adaptations and improvements in the anaerobic power of the players, at the same time of avoiding the negative effects of fatigue.

## Data Availability

The data presented in this study are available on reasonable request from the corresponding author. The data are not publicly available due to privacy reasons.

## Abbreviations

PHV: Peak height velocity
ATP-CP: Alactic anaerobic metabolism
EaS: Early-season
W: Weeks
MiS: Mid-season
EnS: End-season
WL: Workload
AWL: Weekly acute workload
CWL: Weekly chronic workload
ACWLR: Weekly acute to chronic workload ratio
TM: Weekly training monotony
TS: Weekly training stain
RPE: Rating of perceived exertion
RAST: Running-based anaerobic sprint test
RASTmin: RAST minimum
RASTave: RAST average
Fatiguein: Fatigue index
CI: Confidence interval
